# ‘Mens sana in corpore Sano’: Home food consumption implications over child cognitive performance in vulnerable contexts

**DOI:** 10.3389/fpsyg.2022.994399

**Published:** 2022-11-01

**Authors:** Rosalba Company-Córdoba, Michela Accerenzi, Ian Craig Simpson, Joaquín A. Ibáñez-Alfonso

**Affiliations:** ^1^Human Neuroscience Lab, Department of Psychology, Universidad Loyola Andalucía, Seville, Spain; ^2^ETEA Foundation, Development Institute of Universidad Loyola Andalucía, Córdoba, Spain; ^3^Department of Experimental Psychology, Universidad de Granada, Granada, Spain

**Keywords:** children and adolescents’ development, social exclusion risk, diet, cognition, food insecurity, nutrition, rural–urban gap

## Abstract

Diet directly affects children’s physical and mental development. Nonetheless, how food insecurity and household food consumption impact the cognitive performance of children at risk of social exclusion remains poorly understood. In this regard, children in Guatemala face various hazards, mainly related to the socioeconomic difficulties that thousands of families have in the country. The main objective of this study was to analyze the differences in cognitive performance considering food insecurity and household food consumption in a sample of rural and urban Guatemalan children and adolescents at risk of social exclusion. Child cognitive performance was assessed in 134 children and adolescents (age *M* = 11.37; *SD* = 3.54) from rural and urban settings. Language, attention, and executive functions were assessed using neuropsychological tasks. Differences in cognitive performance in each level of food insecurity and household diet consumption were compared using the Mann–Whitney U test. A stepwise multivariate regression analysis was conducted to determine which factors may influence cognitive scores. The results showed that rural and urban groups did not differ in terms of food insecurity. However, considering just rural areas, differences were found between groups with food security and insecurity in attention and executive function tasks. Moreover, differences were found in food consumption for certain groups of food (e.g., meat, *U* = 1,146, *p* < 0.001, *g* = 0.72). Regarding regressions, protein food consumption (e.g., meat and fish), which is related to having a more balanced diet, was a relevant factor in executive performance. Contrary to what we expected, performance in attentional tasks was not related to the consumption of any food group. These findings could help politicians and decision-makers to select actions focused on improving diet balance and food security in families at risk of social exclusion. It is necessary to carry out more specific studies on the factors related to diet that affect the cognitive development of minors at risk of social exclusion. In addition, it is necessary to study the implementation of alternative interventions that include low-cost nutrients, thus ensuring that minors have access to a more balanced diet.

## Introduction

The variety and quantity of food consumed affect children and adolescents’ health and well-being. Usually, children that suffer from nutritional deficiencies are those who live in contexts at risk of social exclusion due to their families’ socioeconomic characteristics. Literature has shown that the cognitive domains that are especially affected in the case of children that live in familiar disadvantaged conditions are language (Huttenlocher et al., 2007), attention (Mezzacappa, 2004; Hackman and Farah, 2009), and executive functions (Noble et al., 2007; Musso, 2010; Arán-Filippetti, 2011). These results have been previously seen in societies with a very high risk of social exclusion, such as Guatemala (Ibáñez-Alfonso et al., 2021).

Regarding difficulties that are associated with the quality and quantity of food consumption, there are two concepts that are important to define. On the one hand, *wasting* is defined as the condition of having a lower weight in comparison to what is recommended for a given height, considering age. On the other hand, *stunting* refers to the condition of having a lower weight than expected to be considered healthy, taking into account age. In a recent study (Yisak et al., 2021), data from 1,534 children were analyzed using linear regression to explore the associations between dietary diversity and early neurodevelopment, with adjustments for the age, sex, and prematurity of the child; the age, sex, and educational level of the caregiver; and family size, income, and simulative care practices and resources. This study reported that 32.4% of children had suspected developmental delays based on the Chinese version of the Ages and Stage questionnaire. According to the United International Children’s Emergency Fund (UNICEF), the number of children who suffer from stunting has declined steadily since 2000 (UNICEF, 2020). Nonetheless, 149.2 million children under 5 were underdeveloped, out of which 45.4 million were suffering from wasting. UNICEF pointed out that more actions need to be implemented in order to improve children’s access to healthy food around the globe. Additionally, diet-related problems are a cause of concern that need to be controlled to achieve the Sustainable Development Goals planned for 2030. Malnutrition costs around 3.5 trillion dollars per year globally (Global Panel on Agriculture and Food Systems for Nutrition, 2016), causing uncountable damage to society. These alarming data should motivate governments about the importance of working together in order to alleviate these conditions in a high number of citizens around the world. Specifically, we cannot forget that we are talking about a group of people in a vulnerable situation that will become the adults that will sustain societies and economies in their respective countries in the future.

One of the challenges low-income families must face is diet imbalance and nutrient scarcity. But how do both factors affect children’s development? The answer to this question is not simple since the mechanisms by which food influences individual development are complex. For sure, adequate nutrition is necessary to ensure a child’s proper bodily and brain development, and the implications that malnutrition and stunting have for children’s growth and general health have been highly studied. Malnutrition refers to imbalances due to deficiency or excess of nutrients, while, as previously stated, stunting is a measure that relates an individual’s weight and height (World Health Organization, 2020). With regard to this, the evidence has shown that severe acute malnutrition, chronic undernutrition, anemia, and iron and iodine deficiency are all conditions that lead to risk factors such as impairments in motor, cognitive, and socioemotional skills development (Prado and Dewey, 2014).

In addition to studying the consequences of malnutrition on a physical level, researchers have also focused on cognitive performance. For example, in a cross-sectional study (Chen et al., 2021) conducted with a sample of 1,293 disadvantaged Chinese preschoolers, of which 33% had anemia, 11% were stunting, and 2% had wasting, researchers obtained relevant results. After controlling for socioeconomic factors (household and parental characteristics), results showed relations between measures of anemia and stunting with children’s cognitive performance scores. In this case, scores obtained in some subscales of the Wechsler Preschool and Primary Scale of Intelligence Instrument, Fourth Edition (WPPSI-IV) by children who suffered from nutritional deficiencies were worse than their counterparts.

Having a balanced diet is especially important in specific periods of development of the nervous system in order to achieve proper socioemotional, motor, and cognitive performance. One example of this are the results obtained in a study in which balanced diet scores were positively related to listening, thinking, reading, writing, math skills, and learning quotient in a sample of 986 children from South Korea (Park et al., 2012). In this regard, it is important to consider that malnutrition and having an imbalanced diet are severe problems for future adults since these conditions in early ages have been shown in a longitudinal study to be associated with attentional problems in adulthood (Galler et al., 2012).

Focusing on the nervous system structure and function, the implications of having a proper and balanced food intake may be obvious. Brain development progress starts soon after conception and experiences rapid growth through a cascade effect (Grantham-Mcgregor et al., 2007). In early years and adolescence, due to the rapid growth of tissues and organs, as well as the body’s needs, the organism is nutritionally demanding. Nutrition influences the trajectory of brain development prior to birth, being an important factor that is initially provided by the mother to the fetus (Morgane et al., 2002; Bhutta et al., 2013). According to studies conducted with animals and humans, certain nutritional hazards during early stages of brain development can lead to disturbances in neural developmental milestones such as axon and dendrite growth, proliferation, and myelinization, among others (Prado and Dewey, 2014).

Both micro- and macronutrients affect children’s development. The most relevant micronutrients for neurodevelopment are zinc, iron, and iodine. For instance, heme iron, the form of iron that is most readily absorbed by your body, is commonly found in meat, fish, and poultry. Iron deficiency is one of the most common problems in the case of children that live in disadvantaged conditions (Perignon et al., 2014), and it is the main cause of anemia. Proper levels of this mineral favor aspects such as myelination and the functioning of dopamine and serotonin systems (Krebs et al., 2017). On the other hand, macronutrients are considered to contribute as a source of energy for the metabolism within the human body (Carreiro et al., 2016). These nutrients are mainly carbohydrates, lipids, and proteins. The intake of carbohydrates, which is associated with increases in glycemic index, may be related to memory and concentration scores in children, but to fully understand how their consumption affects cognitive performance, more research is needed (Wasyluk et al., 2019). Regarding lipids, a systematic review (Taylor et al., 2017) that included interventions focused on nutrition and long-chain polyunsaturated fatty acids supplementation was associated with a marginal increase in crystallized intelligence in children under 10 years. Nevertheless, this effect was not statistically significant (*p* = 0.09). Similarly, protein deficiency has been shown to influence children’s neuroanatomy and neurochemistry since perinatal stages (Chertoff, 2015; Cusick and Georgieff, 2016) as this element is involved in neural formation such as dendritic arborization and cell maturation. For instance, in one study in which cognitive performance was tested in adequately and malnourished children, the results were clear. Protein stunting was especially relevant in the case of higher cognitive processes such as related to executive functioning (cognitive flexibility and working memory), attention, verbal comprehension, memory, and visual perception (Kar et al., 2008). Protein-rich foods include meats, poultry, seafood, eggs, and dairy products.

In another study conducted in India with malnourished boys aged 10 to 12 years, different memory components, associative and conditional learning, were seen to be affected, although motor components were preserved (Agarwal et al., 1995). General development has also been shown to be affected by diet. Using the Age and Stages Questionnaire-version 3 ASQ-3 (Singh et al., 2017) to assess a cohort of children from Nepal, Thorne-Lyman et al. (2019) showed that consumption of animal-based food, vegetables, and fruits was associated with scores in overall development as well as with problem-solving performance (which is considered an executive function). In summary, several studies which have analyzed the impact of food consumption on children at risk of social exclusion have shown that components of executive functioning can be negatively affected. Considering the impact that a nutrient deficit can have on children’s cognition, it is important to know how families consider balancing their diet in terms of quantity and quality of food, and how secure they feel about the familiar nutritional intake. Regarding this, studies conducted in China with samples aged from 6 months to 5 years old have shown that dietary diversity is related with attention, motor, and executive tasks, being balancing diets related with better scores (Li et al., 2021; Zhao et al., 2021).

Some studies have explored how family members report their food insecurity as this factor could be related to different childhood health indicators. Food insecurity, which is the lack of consistent access to sufficient quality and/or quantity of food, generally has six indicators: availability, accessibility, utilization, stability, agency, and sustainability (Food and Agriculture Organization; High Level Panel of Experts on Food Security and Nutrition (HLPE, 2020), and it affects an estimated 800 million people who mainly live in low- or middle-income countries (Food and Agriculture Organization of the United Nations, 2014). This indicator has been shown to be significantly related to children’s health status (Pai and Bahadur, 2020; Gallegos et al., 2021), with risk of illness, anemia, and low-height-for-age ratios being common consequences (Hackett et al., 2009; Anderson et al., 2012; Schmeer and Piperata, 2017). Regarding the risk of illness, in a study that evaluated children whose households reported high levels of food insecurity, Cook et al. (2004) reported that these children were 30% more likely to be hospitalized in comparison to peers with more balanced food intake.

Food-insecure children can also manifest conditions such as anxiety, depression, learning disabilities, aggression, and worse self-perceived health, among other conditions (Casey et al., 2005; Ma et al., 2008). For example, Rose-Jacobs et al. (2008) found that parents from food-insecure households were more likely to report their children’s risk conditions in areas such as language, fine and gross motor skills, and school performance, compared with their counterparts. These consequences in different cognitive processes have been shown to be present from an early age, but they continue over time. Alaimo et al. (2001) and Jyoti et al. (2005) showed that reading, math, and social skills are the areas affected by food insecurity in kindergartners, whereas school-age children suffer the consequences of this condition in aspects of academic performance such as the repetition of the school year or absenteeism, as well difficulties in mathematics. According to the State-of-the-Art review conducted by Gallegos et al. (2021), from a total of 17 studies included in the systematic review in which implications of food insecurity in child development were analyzed, only seven studies examined the implications that food insecurity has in cognitive functioning. The authors of the included studies were focused on outcomes such as reading abilities, math, approaches to learning, cognitive development as well as general cognitive function. These results were congruent with the meta-analysis of de Oliveira et al. (2020), which showed that house food insecurity was associated with lower scores in cognitive outcomes, especially vocabulary and math skills. It is important to consider that despite food insecurity usually being suffered by people who live in low-middle-income countries, most of the research examining this issue has been undertaken in developed countries (Kursmark and Weitzman, 2009; Nord, 2014). As can be seen, we need to understand globally how food necessities perceived by families may influence children’s cognitive development in different ways.

Regarding food consumption patterns, it is important to consider that not all groups of people that live in vulnerable conditions show the same pattern of food consumption as local factors, such as the area of residence, can greatly influence the type and amount of food availability. Considering rural and urban groups, literature has shown that urban populations tend to have better nutritional levels than rural areas (Satria and Mayasari, 2019). For example, in one study, results showed that children and especially girls from urban areas had a greater opportunity to consume more animal-based protein foods (e.g., meat and eggs), while boys from rural areas reported consuming more milk protein items than their urban counterparts (Aziz et al., 2018). Diverse studies conducted in India have shown that sociodemographic and socioeconomic variables, such as being school-aged, low familiar socioeconomic status, low parental education, large family size, and living in rural areas, are significantly related to malnutrition (Siddique et al., 2013; Khan et al., 2015; Parveen et al., 2016). In the case of Latin-American regions, according to an analysis conducted by the Institute of Nutrition of Central America and Panama (INCAP), tomatoes, floured meals (such as “tortillas”), sweets, and beans are among the most consumed food in the case of Guatemalan population (Menchú and Méndez, 2011). In that report, it is possible to see the existence of differences between urban and rural consumption. Differences are also visible depending on the familiar socioeconomic status. For example, the section of the population considered “not-poor” consumes a wider variety of food groups in comparison with their “poor” and “extreme poor” counterparts.

Given the consequences that food insecurity and diet imbalance have on children’s development, one of the main goals of public agents should be the implementation of nutritional interventions that could alleviate the effects of a lack of nutrients in early stages of development. Previous evidence has shown that stunting prevention is useful in the first 1,000 days as the benefits realized from such interventions persist over time. Specifically, cognitive development continues after the first 1,000 days, with earlier and longer lasting interventions being more effective (Black et al., 2015). Specifically in Guatemala, authorities have studied food consumption in the different regions and ethnicities of the country, showing that Maya groups are the most vulnerable in terms of food insecurity (Ramirez-Zea et al., 2014). For example, Gamero et al. (1996), assessed the nutritional intake of 303 pre-school children from rural settings, finding that tortillas and beans were the most consumed food groups. Because of the food imbalance and scarcity, different food interventions carried out in Guatemala have especially focused attention on mothers, children, and adolescents (Leroy et al., 2019). A recent study conducted in this country showed the benefits of a nutritional intervention in terms of food security and dietary diversity in mothers and children from rural areas (Guzmán-Abril et al., 2021). Even though the results were not statistically significant, the improvements found should provide a base to rethink how to design programs to prolong and strengthen these results.

The main aim of the present study is to analyze how sociodemographic and dietary diversity may contribute to explain performance in neuropsychological processes that have been shown to be more impacted in the case of children at risk of social exclusion. Based on the literature reviewed, we formed four hypotheses. Firstly, we hypothesized that families from rural areas would report higher levels of food insecurity than urban families. Secondly, we hypothesized that children whose families reported moderate to severe food insecurity would have lower scores in executive functions and attentional tasks in comparison with the group with a more balanced diet, and that this would be independent of the rural–urban status. Thirdly, we hypothesized that the household food consumption index would be poor to borderline in the case of rural children as opposed to the urban group. Finally, we hypothesized that children from families that can include protein-rich food in their diet (meat/fish, eggs, dairy products) would obtain higher scores in executive and attentional cognitive processes’ tasks.

## Materials and methods

### Participants

The study included participants from one urban and two rural schools. The two rural schools were characterized by the ethnic background of their students, as most of them were indigenous K’iche’. Rural schools had students at risk of social exclusion since their families presented a low socioeconomic status. The urban school was in the suburbs of Ciudad de Guatemala, the nation’s capital city. Students in this school were exposed to community violence as the area surrounding the school is a conflictive point between two criminal gangs. The demographic and food consumption patterns broken down by rural/urban setting regarding the sample are included in Table 1. For each food group, the median number of days per week the food group was consumed, as well as the range, are included.

**Table 1:** Demographic and Food Consumption Patterns Broken Down by Rural/Urban Setting.

		**Rural(*n* = 94)**		**Urban (*n* = 40)**	
**Measures**	**Range**	***M***	***SD***	***M***	***SD***
Age	6–17	10.65	2.95	13.07	4.21
MLPE	−	6.41	5	6.69	3.68
Income (GTQ)	−	1582.85	1624.74	2144.44	1555.22
People living in same household	−	6.78	2.29	5.36	1.66
*ELCSA*	0–15	4.7	4.19	3.28	3.76
*HFCS*					
Tortillas	0–7	6.35	1.47	6.15	1.47
Beans	0–7	3.42	2.1	3.95	2.19
Vegetables	0–7	4.48	2.34	4.6	2.14
Fruits	0–7	2.95	2.42	3.83	2.14
Meat and Fish	0–7	2.07	1.75	3.4	2.04
Oils	0–7	2.88	2.37	4.92	2.17
Roots	0–7	2.73	1.88	2.97	1.99
Eggs	0–7	3.7	2.28	4.63	2.36
Dairy products	0–7	2.31	2.48	3.93	2.36
Sugar	0–7	4.98	2.58	5.13	2.49
*FCS*	−	68.25	29.72	86.92	31.75

MLPE, Mean Level of Parental Education (in years); GTQ, Guatemalan Quetzals; ELCSA, Latin-American and Caribbean Latin-American food security scale; HFCS, Household Food insecurity Consumption Scale; FCS, Food Consumption Score.

All participants had an IQ higher than 80 assessed by the non-verbal intelligence test TONI-II (Brown et al., 2009). In Table 1, demographic data along with statistics (mean and rank) of days in which each food group is consumed are also included. From a total of 154 participants, 20 refused to answer this questionnaire, and so analyses were conducted on a total of 134 participants (age *M* = 11.37; *SD* = 3.54; 70 females). 26.4% of the sample were considered to be indigenous K’iche’. Concerning food insecurity, the general mean score of ELCSA scale was *M* = 4.29 (*SD* = 4.01) which means that the general level of food insecurity was mild (Table 1). Regarding the FCS, the global score was acceptable, 73.77.

### Procedure

In an orientation session prior to the commencement of any assessment, the research team explained to teachers and parents the aim and the testing procedures. Parents were asked for permission for their children’s participation by signing informed consent. After parents had accepted the enrollment of their children into the study, they completed both sociodemographic and socioeconomic questionnaires to determine some family socioeconomic indicators. Each child was assessed individually by local trained psychologists in a single session lasting 2 h in a school classroom with adequate light and ventilation. The order in which cognitive instruments were administered was randomized. Since the assessment was long, especially for younger children, sample rest time was allocated during the testing session. This study was approved by the Andalusian Biomedical Research Ethics Committee.

### Questionnaires

#### Latin-American and Caribbean Latin-American food security scale (ELCSA)

This questionnaire has been validated in the Guatemalan population after its application in a study by the Food and Agriculture Organization of the United Nations in 2011 (Ballard et al., 2013), showing good psychometric properties. In that study, it was shown to be correlated with sociodemographic variables related to poverty. This questionnaire was answered by the parents or caregivers who were asked about how safe they feel in relation to the food they can provide to the family. Scores range between 1 and 15 with higher scores reflecting higher food insecurity. The total score is interpreted as follows: 1–5, mild; 6–10, moderate; and >10, severe insecurity. In a previous study (Company-Córdoba et al., 2020), this scale showed good reliability, Cronbach’s α = 0.85, and Guttman’s split-half reliability = 0.80.

#### Home food consumption score

This questionnaire assesses how many days per week the family consumed certain types of macronutrients (e.g., vegetables, meat, and fish) in the past week (Consorcio de Organizaciones Humanitarias, 2019), and is used as a proxy measure of household food access. This scale reports the *Food Consumption Score* FCS (Consorcio de Organizaciones Humanitarias, 2019), which is accepted as a good measure of diet quality since it considers the nutritional importance of each group of food, with higher scores indicating better nutrition. The different food groups have been assigned the following values: Meat and fish (4); dairy products (4); eggs (3); legumes and nuts (3); cereals (2); roots and tubers (2); vegetables (1); fruits (1); oils and fats (0.5); and sugary foods (0.5). The total score is then assessed, and interpretation is as follows: Poor (0–21); borderline (21.5–35); and acceptable (>35).

### Instruments

The following neuropsychological instruments mainly assess certain cognitive processes. Nonetheless, it is important to point out that it is impossible to strictly dissociate different processes when assessing cognitive abilities. In a previous study with the same sample (Company-Córdoba et al., 2020), the following scales have shown good reliability.

#### Peabody picture vocabulary test, PPVT-III

The PPVT-III is used to assess the receptive vocabulary in individuals aged 2 to 90 years (Dunn et al., 2010). Although the use of this tool in the case of indigenous population has been questioned due to the cultural biases inherent in the stimuli (Leigh and Gong, 2008; Haitana, 2010), the PPVT version used in this study has been shown to have adequate psychometric properties (interclass coefficient 0.95, *p* < 0.001; Rivera and Arango-Lasprilla, 2017) and there is specific normative data for the Guatemalan pediatric population (Rivera et al., 2017). The tool has 192 items in total, each one consisting of a sheet with 4 illustrations that show different images related to a concept. The task is to select the illustration which best fits the meaning of a concept indicated by the examiner. This test has shown good internal consistency (Williams and Wang, 1997) and good concurrent validity (Hayward et al., 1997). The original validation study indicated Cronbach’s α > 0.90 (Henninger, 2011). The maximum possible score is 192. The required time for the assessment is about 10–15 min.

#### Shortened version of TOKEN test, TOKEN

This version of Token test allows the assessor to measure the number of understood commands without redundancy (De Renzi and Faglioni, 1978). The tool is composed of 20 tokens in five colors (yellow, red, black, white, and green), two sizes (big and small), and two shapes (circle and square), and the answer document. This reduced tool has 36 commands, which the child has to perform, divided into six parts. Its construct and concurrent validity have been shown to be acceptable when used with children (Gallardo et al., 2011) and the computerized version also has good psychometric properties (McNeil et al., 2015). The internal reliability has been reported as 0.92 (Strauss et al., 2006). Test–retest reliability has shown values of 0.75 (Rivera and Arango-Lasprilla, 2017). Its administration takes around 10 min.

#### Phonological and semantic verbal fluency tests

In this study, the test was administered and scored following Olabarrieta-Landa et al. (2017) guidelines. The test includes phonological and semantic verbal fluency modalities, which we have combined by taking the average of the two scores to obtain a single measure of verbal fluency. The scores depend on the number of words the participant can emit in each category in 60 s. This test has shown acceptable levels of test–retest reliability in previous studies (Preston, 2010), in both phonological and semantic subtests (Rivera et al., 2017). Its administration takes around 8 min.

#### Symbol digit modalities test, SDMT

In this task, the participant must replace a series of figures with other numbers by using a previously learned key (Smith, 2002). The final score is the total number of correct substitutions made in 90 s. The maximum score is 110. Its administration can be verbally or written. The latter option was selected for this study. This test has shown very good test–retest reliability (ICC = 0.80; Smith, 2002), and its convergent validity has also shown to be good with other cognitive measure tests (Strober et al., 2019). The symbol digit modalities test assesses neurocognitive functions such as attention, speed processing, or working memory. This task has a duration of 2 min.

#### Concentration endurance test, d2

The d2 assesses different abilities related to attentional skills (e.g., speed and amount of performance, quality of performance, and the relationship between speed and accuracy of attention (Brickenkamp, 2009). This test has 14 lines with 47 items on each line. Participants have 20 s to respond to each line. The participant must mark the three items that the examiner has previously shown. Although the instrument has different scores (e.g., total responses, total correct responses, omission and commission errors, effectiveness of the performance, and concentration index), in this study, only the concentration index was used due to its relevance to the research objectives. The d2 has shown good psychometric properties with values of internal consistency for Cronbach’s. α of approximately 0.95 and a validity coefficient of Cronbach’s α = 0.47 (Bates and Lemay, 2004; Brickenkamp et al., 2013). Its administration takes around 10 min.

#### Modified Wisconsin sorting card test, M-WCST

This test is mainly useful to assess some executive function processes such as working memory, attentional flexibility, and response inhibition (Schretlen, 2010). This version has a total of 48 response cards and four key cards with different shapes, colors, and number of stimuli on them. Each time a card from the deck is taken, the participant has to select which category the card belongs to. The participant must discover a new association rule every sixth card. The WCST has shown great reliability in perseverative and non-perseverative error scores (between 0.91 and 0.96; Heaton, 1981) and good test–retest reliability (Ozonoff and Mcevoy, 1994), showing test–retest values of 0.80 in the correct categories score (Rivera and Arango-Lasprilla, 2017). The administration of this test takes around 15 min.

#### Trail making test, TMT A-B

This test consists of connecting dots in a logical order with the TMT-A being comprised of numbers and the TMT-B comprised of numbers and letters (Reitan and Wolfson, 1985). In this study, we only consider form B. The time needed to complete the task is the score for this instrument. Both forms of TMT have shown good reliability and validity scores (Giovagnoli et al., 1996; Arbuthnott and Frank, 2000) and good test–retest values in both subtest TMT-A (0.68) and TMT-B (0.76; Rivera and Arango-Lasprilla, 2017). Its administration time depends on the participant’s performance, but it is usually around 5 to 10 min.

#### The Stroop color and word test

The Stroop test is used to assess response inhibition due to interference (Golden, 2010). This test has three pages with 100 items on each page. The first part has “red,” “green,” and “blue” written on it in black ink. The second part has 100 meaningless items printed with different colors (red, green, or blue). The last part has the same number of elements, but this time they are color words printed with different colors. The total score is the number of words the participant can read in 45 s for each trial. With this task participants obtain a resistant to interference score. Psychometric properties of this test have been shown to be good in terms of validity, and have a reliability of around 0.70 in all subtests (Barreto et al., 2016). This test provides a score that indicates how well the participant can resist interference. Stroop test administration time is approximately 5 min.

### Statistical analysis

Statistical analyses were conducted using SPSS version 28 (IBM Corp, 2021). Sociodemographic and socioeconomic variables are presented in Table 1. A Chi-square test revealed no significant differences between groups in terms of gender, χ^2^ (1) = 0.269, *p* = 0.604. Firstly, we assessed if the rural group showed higher levels of food insecurity compared to urban ones. Secondly, we assessed if children whose families reported moderate to severe food insecurity had lower scores in executive functions and attentional tasks in comparison with the group with a more balanced diet. Thirdly, regarding the household food consumption score, we evaluated if the index was lower in the case of rural children in comparison with the urban group. Finally, it was hypothesized that children with a more varied diet (e.g., can include proteins in their diet) will obtain higher scores in executive and attentional cognitive processes’ related tasks. Due to departures from normality in some distributions, Mann–Whitney *U* tests were conducted to detect group differences for the first three research questions. To assess the final research question, percentiles obtained in different cognitive tasks were considered when analyzing differences between the living environments (urban vs. rural), along with levels of food insecurity. Standardized percentile scores applicable to the Guatemalan pediatric population for all of these instruments have previously been published (for a detailed description, see Ibáñez-Alfonso et al., 2021). The process for converting the raw scores to percentile scores considers factors such as age, gender, and mean years of parental education. Accordingly, as we analyzed percentile scores, it was not necessary to control for these factors as they were already accounted for at the point of converting the raw scores to percentile values. Since variables such as age, gender, and mean years of parental education were controlled using percentile scores, these variables were not included as predictors in the multiple regression analyses. Thus, the stepwise multivariate regression analyses included scores in food insecurity (ELCSA), Food Consumption Score weighted index (FCS), and food groups incorporated in the Household food consumption scale (HFCS).

## Results

The Mann–Whitney *U* tests were conducted to offer comparisons between rural and urban groups (Table 2). The ELCSA score did not differ significantly between urban and rural settings (*U* = 2,750; *p* = 0.181; g = −0.338). However, significant differences between regions were found in consumption for five out of 10 food groups. Lower scores, reflecting a poorer diet, were obtained by rural children in all cases.

**Table 2:** Comparisons between urban and rural children in relation to food consumption and cognitive performance.

**Measures**	**Range**	**Rural (*n* = 94)**	**Urban (*n* = 39)**			
		***Median (Rank)***	***Median (Rank)***	***U***	***p***	***Hedges’ g***
Age	6–17	11 (11)	15 (11)	1,179	<0.001	1.09
MLPE	−	5 (17)	6 (13)	1,598	0.169	0
Income (GTQ)	−	800 (6925)	1,550 (6000)	729.5	0.004	0.33
People living in same household	−	7 (11)	5 (7)	1704	0.003	−0.55
*ELCSA*	0–15	4 (14)	2 (15)	2,156	0.106	−0.27
*HFCS*						
Tortillas	0–7	7 (7)	7 (4)	2122.5	0.100	0
Beans	0–7	3 (7)	4 (7)	1,591	0.181	0
Vegetables	0–7	5 (7)	5 (7)	1796.5	0.677	0
Fruits	0–7	2 (7)	3 (7)	1405.5	0.019	0.49
Meat and Fish	0–7	2 (7)	3 (7)	1,146	<0.001	0.72
Oils	0–7	3 (7)	5 (7)	962	<0.001	0.99
Roots	0–7	2 (7)	3 (7)	1655.5	0.265	0
Eggs	0–7	3 (7)	5 (7)	1,451	0.034	0.49
Dairy pr.	0–7	1.5 (7)	3 (7)	1,106	<0.001	0.49
Sugar	0–7	7 (7)	7 (7)	1818	0.744	0
*FCS*	−	65 (119)	82.5 (119)	1205.5	0.002	0.61
*Neurops. PC*						
PPVT-III	0–100	61 (100)	65.5 (95)	1690.5	0.406	0.18
Token	0–100	72.4 (99)	54.59 (96.2)	2,312	0.036	−0.41
Phon.fl/a/	0–100	52 (79)	45 (89)	1947.5	0.668	−0.11
Sem. Fl. ani	0–100	55.5 (98)	38 (95)	2300.5	0.041	−0.41
SDMT	0–100	61.7 (97.7)	61.4 (90.6)	1830	0.640	−0.11
d2 CON	0–100	52 (99)	61.5 (86)	1,278	0.016	0.49
M-WCST cor.	0–100	66 (92)	26 (96)	3082.5	<0.001	−1.41
M-WCST pers.	0–100	67 (92)	53.5 (93)	2,359	<0.001	−0.59
TMT-B	0–100	71 (97)	35 (95)	1955.5	<0.001	−0.95
Stroop interf.	0–100	37 (96)	49 (95)	1230.5	0.508	0.16

MLPE, Mean Level of Parental Education (in years); GTQ, Guatemalan Quetzals; ELCSA, Latin-American and Caribbean Latin-American food security scale; HFCS, Household Food insecurity Consumption Scale; FCS, Food Consumption Score.

### Food insecurity analysis

The general results showed that 24.8% of families consider themselves to be secure, 39.1% consider themselves to have mild food insecurity, 26.9% believe that they have moderate food insecurity, while 9% of families reported having severe food insecurity. The specific results are included in Table 3. The mean score of rural and urban groups in terms of ELCSA did not differ significantly.

**Table 3:** ELCSA ranks by group.

	**General**	**Rural (*n* = 94)**	**Urban (*n* = 39)**
**ELCSA ranks**	***n***	***n***	***n***
Severe	12 (9.02%)	10 (10.6%)	2 (5.13%)
Moderate	36 (27.07%)	29 (30.85%)	7 (17.95%)
Mild	52 (39.10%)	33 (35.11%)	19 (48.72%)
Secure	33 (24.81%)	22 (23.4%)	11 (28.20%)
N/A	1	0	1

N/A, No answer.

Differences in cognitive performance between rural and urban groups were analyzed by firstly considering the ELCSA sub-groups separately (secure, mild, moderate, and severe). Results showed that there were significant differences in executive functioning and verbal comprehension between rural and urban dwellers in the secure group, along with significant differences in attentional and executive functions between rural and urban dwellers in the mild group. Rural and urban families that reported having moderate food insecurity showed significantly different scores in Semantic fluency and M-WCST tests. Additional Mann–Whitney *U* tests were conducted to compare cognitive scores among groups in a more detailed way. In the tables presented in the Supplementary materials, the secure group was compared with the mild, moderate, and severe groups. As can be seen in Supplementary material (TS1), there were significant differences in the general group scores, these were due to the security and the rest of food insecurity groups from the rural areas in the case of attention and executive functioning tests.

### House food consumption score analysis

The mean Household Food Consumption Score for the entire sample was *M* = 77.59 (*SD* = 30.73). This score suggests that the sample experienced acceptable household food consumption. Even when household food consumption was examined within ELCSA food security, no group experienced a poor or borderline food consumption index, since all groups scored >35 (Table 4). Nonetheless, the severe food insecurity group of participants from the rural group obtained the lowest score overall participants (*M* = 46; *SD* = 22.33).

**Table 4:** Household Food Consumption Score reported in each Food Insecurity group.

	**ELCSA food insecurity group**				
	**General**	**Secure**	**Mild**	**Moderate**	**Severe**
	M (SD)	M (SD)	M (SD)	M (SD)	M (SD)
Rural	68.23 (29.7)	93.2 (27.1)	66.9 (24)	58.16 (28.1)	46 (22.3)
Urban	86.9 (31.8)	87.4 (25.1)	93.4 (37.7)	68.08 (18.6)	67.3 (21.6)
Total FCS	73.8 (31.4)	91.3 (26.2)	76.6 (32.1)	59.91(26.7)	49.5 (22.8)

FCS, Food Consumption Score.

Regarding the food groups included in HFCS, as shown in Table 2, food consumption by food groups was significantly different in fruits, meat and fish, oils, eggs, and dairy products with resulting *p*-values ranging from <0.001 to 0.034.

As can be seen in Table 5, the consumption of vegetables, roots, and tubers was significant in the case of language tests (*p* < 0.05). Furthermore, the consumption of foods with high protein content, such as beans, oils, and meat as well as dairy products, was significantly related to executive functioning processes in the case of M-WCST correct responses, perseverative errors, and TMT-B scores. Food group consumption was not significant in any case when considering attentional-related neuropsychological tools (SDMT and d2 concentration index).

**Table 5:** Multivariate regression analyses predicting performance in 10 neuropsychological tasks based on food security (ELCSA).

**Neuropsychological tests**	**Predictors**	**B**	**Standard error**	**β**	***t***	***value of p***	**Adjusted R** ^**2**^
PPVT-III	–	–	–	–	–	–	–
Token	–	–	–	–	–	–	–
Phonol. fluency /a/	Constant	51.48	1.62	–	31.83	<0.001	0.030
	Vegetables	3.63	1.63	0.193	2.22	0.028	–
Sem. fluency, animals	Constant	51.06	2.38	–	21.49	<0.001	0.039
	Roots /tubers	−5.93	2.37	−0.215	−2.5	0.014	–
SDMT	–	–	–	–	–	–	–
d2 concentration index	–	–	–	–	–	–	–
M-WCST corr. Cat.	Constant	51.27	2.3	–	22.39	<0.001	0.108
	Beans	−6.74	2.4	−0.245	−2.81	0.006	–
	Oils	−7.85	2.73	−0.282	−2.87	0.005	–
	Meat	5.51	2.74	0.198	2.01	0.046	–
M-WCST pers. err	Constant	54.58	2.54	–	21.5	<0.001	0.053
	Beans	−7.26	2.54	−0.245	−2.86	0.005	–
TMT-B	Constant	56.23	2.87	–	19.59	<0.001	0.053
	Beans	−8.32	3.11	−0.270	−2.67	0.009	–
	Dairy products	6.68	3.25	0.207	2.05	0.043	–
Stroop interference	–	–	–	–	–	–	–

Rows containing only dashes indicate that the overall regression was not significant, and as such, no predictor was related to the neuropsychological ability assessed. FCS, Food Consumption Score; HFCS, Household Food insecurity Consumption Scale.

## Discussion

The general objective of this study was to explore how food insecurity and balanced household food consumption would impact the neuropsychological performance of a sample of Guatemalan children at risk of social exclusion. In order to do so, we analyzed differences in cognitive performance in terms of food insecurity and food consumption in participants coming from rural and urban settings.

Firstly, we hypothesized that children from rural areas would present significantly higher scores in food insecurity due to the low reported family income. We based this assumption on the fact that both food security and availability are related to familiar economic conditions. Contrary to expectations, results showed that there were no significant differences between rural and urban groups in this index. However, we note that a similar result has been also reported in studies conducted with rural and urban school children in Peru and Colombia (Aquino et al., 2014; Bermúdez et al., 2020). Furthermore, in a study conducted with a sample of American Indian families, urban rather than rural groups exhibited higher levels of food insecurity (*p* = 0.05; Tomayko et al., 2017). One reason that could explain this last result is that although rural families reported lower economic resources, they also tend to live closer to the source of the food, and so can access some foods directly, whereas urban populations rely more on purchasing their food supplies. General cost of living also tends to be higher in urban areas, thus reducing the benefit of having more economic resources. On average, the level of food insecurity experienced by families in the present study was mild. That said, it is important to consider that most parents assessed in this study did not feel that their families were secure in terms of food quality and quantity.

Considering levels of food insecurity, the analysis showed that there were significant differences between secure rural and urban groups in language comprehension and executive performance, as well as differences in executive and attentional scores in mild food insecurity groups. In the case of moderate groups, there were differences in semantic fluency score and M-WCST correct answers. No differences were found in insecure groups. These results partially confirm our initial hypothesis since we supposed that children with middle levels of food insecurity would present more differences in terms of cognitive performance. While secure families have better socioeconomic conditions than insecure families, in this study it is notable that rural children showed a better cognitive performance than the urban groups. This leads us to think that apart from economic factors, there may exist other variables that influence cognitive performance in children that may have access to minimum needs. Similarly, children whose families reported severe food insecurity may have worse economic conditions, and their context of deprivation could be more homogeneous (rural and urban).

Secondly, we hypothesized that children whose families reported severe food insecurity would have lower scores in attentional and executive tasks, especially when being compared with the security group. As was shown, for the two attentional tasks included, when comparing just rural families, percentile scores in SDMT were significantly different between secure and severe insecurity families (*U* = 41; *p* = 0.036), as well as when comparing secure and mild insecurity families (*U* = 218; *p* = 0.023). Previous evidence has shown that food insecurity could be related to Attention-Deficit Hyperactivity Disorder (ADHD) symptoms in children, although more studies are needed in relation to this conclusion (Lu et al., 2019). Nonetheless, a study conducted in low-income neighborhoods in Quito (Ecuador) failed to find significant relations between household food insecurity and attentional scores in children from 6 to 12 years (Weigel and Armijos, 2018).

Results regarding executive functioning were in the line with the findings in previous studies. Specifically, scores in M-WCST perseverative errors (*U* = 37, *p* = 0.002) and TMT-B (*U* = 42.5, *p* = 0.041) in the rural group were different when comparing the secure with the severe food-insecure groups. There were also significant differences between secure and mild food insecurity groups in the case of the rural group in M-WCST perseverative errors score (*U* = 179.5, *p* = 0.002). Other studies that assessed children at risk of social exclusion have found relations between presenting a low food insecurity score and having problems managing executive skills such as self-control (cognitive inhibition) and working memory (Grineski et al., 2018). When assessing executive abilities of a sample of children included in the Head Start program, results showed that the food environment was significantly related to executive function scores, specifically, better familiar food environment was related to better executive function (Bryant et al., 2020).

Additionally, we found differences in the case of the percentile scores obtained by the secure and mild groups in the Token test (language comprehension). Although this result was not contemplated in the initial hypothesis, it is interesting to consider if language scores could be affected by food insecurity in the case of rural children (*U* = 217, *p* = 0.012). Nonetheless, this result is congruent with that found in a cross-sectional study conducted in Port Elizabeth, South Africa (Beckmann et al., 2021), in which higher food insecurity scores in girls were associated with their lower scores in language (*p* < 0.05).

Thirdly, regarding Household Food Consumption Score, we hypothesized that families from rural areas would present a food diversity score from poor to borderline that would be significantly different from their urban counterparts. Literature has shown that children who grow up in urban settings could access a more balanced diet (Satria and Mayasari, 2019). The results of the present study showed that both rural and urban showed an acceptable index. Nonetheless, comparing urban and rural areas in terms of food insecurity revealed that, lower food diversity scores were obtained by the severe insecurity rural group and the only value considered to be “poor” in the whole sample came from a participant living in a rural setting.

Considering the food balance in a detailed way (Table 1), families in both urban and rural settings showed differences in weekly consumption in the case of fruits, eggs, oils, meats, and dairy products (the last three *p*s < 0.001), and this is in accordance with the findings in other studies (Parveen et al., 2016; Aziz et al., 2018; Satria and Mayasari, 2019). Thus, one of the conclusions obtained from this comparison is that products that may be more expensive in Guatemala could be less affordable for the group that shows more economic difficulties. This is in line with the results obtained by the INCAP in 2006 (Menchú and Méndez, 2011), that reported the most consumed food groups by the Guatemalan population coincide with those consumed in the present study. As can be seen, the food groups in which this difference is more evident are nutrients that could be difficult to obtain in the case of the rural population in Guatemala, not only in terms of the direct availability of them, but also with respect to affordability. Cheaper food groups seem to be similarly consumed by rural and urban families (e.g., flours, vegetables, etc.). The case of sugary food is somewhat surprising as it seems to be similarly consumed by both groups. According to the data, both groups consume sugary products daily, with these findings being in line with the observed by the INCAP analysis (Menchú and Méndez, 2011). For example, the INCAP data showed that white sugar was consumed in more than 75% of families in both rural and urban areas. It would be interesting to analyze the reason why this product is highly consumed in this country, but probably the low cost and easy accessibility would explain its relatively frequent consumption. This is already an issue of concern in the country since obesity is an increasing problem. More generally, studies conducted with schoolchildren have shown negative correlations between the consumption of sugary healthy food groups (e.g., sweet snacks, or sweetened beverages and sweetened desserts) and academic achievement in math and English scores (Bleiweiss-Sande et al., 2019), as well as with thinking, listening, and writing abilities among others cognitive skills (Park et al., 2012). One of the actions proposed by the government is to increase the price of sugary beverages, but this is still yet to be implemented.

The food groups least consumed by the rural group were animal proteins and dairy products, with this pattern of consumption similar to that reported by rural indigenous people from México (Cordero-Ahiman et al., 2017). Regarding food diversity, better scores in this factor have been shown to be related to better academic achievement in previous studies (Beckmann et al., 2021).

Finally, we also hypothesized that the reported weekly consumption of protein-rich food would be more related to the performance of attentional and executive tasks. Consisting of animal protein, these food groups may not be affordable for many families in Guatemala. Hence, children that have access to these kinds of proteins supposedly will have a more balanced diet. Generally, regarding the food groups that may be related to cognitive performance, as can be seen in Table 5, the consumption of high-protein food groups was significantly related to executive functioning. This confirms our initial expectations. These results are congruent with previous literature which had studied protein-deficient groups of children in terms of executive performance in processes such as information processing or problem-solving, among others (Kar et al., 2008; Stein et al., 2008; Thorne-Lyman et al., 2019). The reason to expect this was not due to the protein consumption *per se*, but the fact that families that can afford these kinds of food groups would probably have a more balanced diet. A point related to this is that, although beans are a high-protein content food, it is not an “expensive” product in the country. Furthermore, it is remarkable that, even though protein consumption is usually assessed considering animal source food such as meat, fish, and eggs, beans are also nutrients which have a high proportion of vegetable proteins. Thus, beans are a source of micronutrients such as vitamins, iron, folic acid, zinc, calcium, phosphorus, and potassium. Actually, beans are in one of the food categories (legumes) most consumed by Guatemalans according to the report presented by the INCAP in 2011 (Menchú and Méndez, 2011). In the regression analyses, the consumption of this product was negatively related to executive functioning scores. This nuance should be considered, and more detailed studies should be done in this regard.

Results regarding the implication of food groups in attentional tests (SDMT and d2 concentration index) were not especially significant in the case of the assessed sample. This was opposite to what we expected since other studies have reported this result (Kar et al., 2008; Kaur and Sharma, 2019). This relation has been previously studied in children from rural Guatemala. Two groups were given supplements of atol (a protein-enriched beverage) and “Fresco” (non-protein-enriched beverage) and cognitive measures were taken before and after consumption. Results showed that children who consumed atol had better scores in reaction time, among other measures, in comparison to their counterparts (Pollitt et al., 1993). In another study conducted on a sample of Indian children who attended governmental and private schools, food consumption was analyzed, with the results showing that the consumption of dairy products among others was positively related to attention span (*p* < 0.05) and memory tasks (Kaur and Sharma, 2019). The importance of a balanced diet, as pointed out in the introduction, is evidenced by the fact that it has been found that minors presenting imbalances during childhood have worse attentional scores in adulthood (Galler et al., 2012).

Some food groups, such as fruits, did not seem to especially influence executive scores as initially expected. Nonetheless, vegetables were a significant influence on SDMT (attention, *p* = 0.035) and M-WCST correct responses (executive, *p* = 0.033), with this latter being an inverse relation. Other studies have shown relations between vegetable consumption and better cognitive skills (Kaur and Sharma, 2019; Thorne-Lyman et al., 2019). These results are partially congruent with those obtained in a study conducted in a sample of 353 participants aged 9 years, in which weekly vegetable and fruit intake did not correlate with executive performance scores assessed with the Behavior Rating Inventory of Executive Function (BRIEF; Riggs et al., 2010). One possible interpretation is that children who consume a higher number of vegetables could receive more home-made meals rather than processed food.

As was explained in the introduction, the INCAP has described general food consumption patterns of the Guatemalan population (Menchú and Méndez, 2011). In that report, floured meals, sweets, and beans were the most consumed food groups. As that report pointed out, more advantaged families have access to a more varied diet, and differences were reported between urban and rural food consumption patterns. Focusing on protein consumption, it is possible to see that egg consumption is slightly higher in rural areas (but 75% of rural and urban households consume them), while there is a difference in meat consumption between areas. Meat consumption is common in 75% of urban households, but only in 50% of rural households. In this regard, it is necessary to consider that in the present study 66.7% of the sample is indigenous. Indigenous populations are usually characterized by living in rural areas, suffering from poverty and social exclusion. Then, it is important to consider that, in the case of children who have less economic resources, it is difficult to know if worse scores in certain tasks are a consequence of having fewer opportunities to consume proteins, or, are due to the fact that these children probably lack the access to a more balanced diet. That is, in the group that is economically more disadvantaged, the more advantaged subgroup probably will have a more balanced diet, which will, in turn, be related to better general development and hence, better cognitive performance. These issues could be resolved in future studies in which more specific data about familiar dietary paths will be offered.

## Limitations and future studies

Although the results presented in the present study are interesting, there are some limitations that must be noted. Firstly, it is important to consider that the instrument used to assess weekly diet consumption is self-reported. The responses given about food group consumption may be influenced by social desirability. Parents could modify their responses to avoid possible repercussions. It is important to take into account the fact that a high percentage of the sample are indigenous, and some conditions are concomitant in their case (e.g., rurality, bilingualism, or poverty). It would be necessary to establish a comparison with a non-indigenous sample to better understand how factors are related to household food intake.

Related to the questionnaire used to assess household dietary diversity, it is important to consider that the instrument asks how many days per week a family consumes a certain food group, but this questionnaire is not considering the portions of food the children are consuming or how many times per day they are eating. For example, extremely small portions of relevant food groups that are consumed several days per week are probably not sufficient to avoid inadequate levels of required nutrients. Moreover, this instrument does not take into consideration the case of vegetarians or vegans, who have access to certain micro and macronutrients exclusively through a plant-based diet.

Another limitation comes from the questionnaire used to assess food intake. It is difficult to classify all food into just a few groups, and likely there are other nutrients that may not be included that have gone unnoticed in this study. Since we assessed a sample of Guatemalan children (rural and urban), maybe we are not aware of other nutrients that they consume daily. An example of this is “atol.” Atol is a drink made with flour and water that the rural educational centers provide to students to reinforce food intake in these infants. We have no information about other supplements taken. Families were asked about consumption within the household, therefore food that children received externally was not accounted for. Thus, the obtained results must be interpreted with caution. In future studies, it would be appropriate to use a more complete household food consumption questionnaire (considering micro and macronutrients) created with the help of locals with an intimate knowledge of the assessed population’s consumption habits. In fact, due to the large number of indigenous Maya participants, it would be necessary for future studies to consider how this population particularly perceive and classify food groups, since culture also affects food knowledge and consumption (Cuj et al., 2020). Another point to take into account in the case of the food intake questionnaire is that it only considers food intake in the previous week, so the information provided in this regard is quite limited. In order to address this shortcoming, it would be necessary to include food intake questionnaires that consider more extended periods of time in order to have a more complete picture of the diet balance in these children.

A further limitation is that as we only assessed children over 6 years old, we have no information regarding infant diet. According to Black et al. (2015), much of brain development occurs from birth until the age of 5. Potentially, this means that our cognitive data was influenced, at least partially, by previous dietary patterns not captured in our study. For this reason, knowing more about the first 5 years of these children’s lives would improve our ability to determine the variables that may influence their cognitive development.

In this regard, interventions based on food or supplement implementation are actions that have been carried out to improve the health of children at risk of social exclusion. After analyzing the results obtained, we propose to find a way to undertake interventions in these populations based around including protein-enriched food in their diets that are easy to maintain over time and are also low in cost. An example of this is the case of protein-enriched legumes, grains, or seeds (e.g., beans, peas, spelt, oats, cornmeal, rice, etc.). In this study, the consumption of beans resulted in significant differences in executive function scores. These types of legumes (vegetable source of protein) are more satiating than animal protein sources. The fact that they are satiating can influence the negative consequences of hunger and may have consequences on executive performance (Kristensen et al., 2016).

Moreover, in future studies, it would be interesting to analyze the diet balance and food insecurity by considering factors such as age, gender, or ethnical group to obtain a clearer picture of how these factors may affect individuals. For example, the need for certain types of nutrients at different life stages would allow for a better understanding of how the specific composition for what is considered to be an adequate diet could vary for children and adolescents. In the case of gender, it would be interesting to determine if diet needs to vary at important periods of development, such as in the case of menarche for girls. To conduct those studies, it would be necessary to recruit a larger sample to obtain valid data of sub-groups analysis.

A final, but important limitation is the fact that ethnicity and urban–rural status were confounded in our sample. Of the 89 indigenous K’iche’ participants, just 2 lived in an urban environment. Similarly, of the 61 non-indigenous participants, just 8 lived in rural areas. This is unsurprising in the sense that it reflects the reality of the demographic breakdown in Guatemala; by and large, indigenous families live in rural settings and non-indigenous families live in urban settings. Nevertheless, it is important to note that the children with an indigenous background had, on average, 2 years less education than their urban counterparts (*p* = 0.013), and the mean level of parental education (MLPE) was also lower in the indigenous group (mean difference of 2 years, *p* = 0.007). Accordingly, we reran some of our analyses comparing indigenous and non-indigenous children, controlling for age and MLPE. The 2 groups did not differ significantly in the food security scale (ELCSA, *p* = 0.168), but they did differ in the consumption of certain types of macronutrients: indigenous children ate fewer fruits, meat and fish, dairy products, eggs, and oils and fats than non-indigenous participants (*ps* ≤ 0.017). Regarding neuropsychological performance, we found a very similar pattern to the results presented in Table 2 (comparing rural and urban dwelling children), and this is no doubt due to the confounding between area and ethnicity (a comparison of cognitive performance by participant ethnicity is presented in TS4 at Supplementary material). Complementary, as the multivariate regression analyses predicting performance in the 10 neuropsychological tasks shown in Table 5 did not make a distinction between rural/urban participants, considering the sample as a whole, results can be interpreted in a similar way between indigenous/non-indigenous participants due to the overlapping of the variables area of residence and ethnicity. In summary, it is not possible to separate area from ethnicity in the present study, and the conclusions reached about rural vs. urban dwellers are very similar to what was found when the data was reanalyzed from the point of view of ethnicity. The only way to disentangle these two factors would be to undertake a new study which involved targeted recruiting to ensure that there was an even distribution of participants across the four conditions that result when these two factors are combined.

The goal of agents interested in this relevant issue should be to find a way to take advantage of local and available resources so that families living in disadvantaged conditions can feed their children in the best possible way at minimum costs. The intake of a balanced diet favors both physical and cognitive development, this being a basic principle for the well-being of thousands of children. In helping to achieve this goal, the scientific community, and groups responsible for ensuring children’s well-being would be acting in line with the Sustainable Developmental Goals included in the United Nations’ Agenda for 2030. Finding a way of helping these communities by elaborating plans that favor the fulfillment of these goals is a responsibility of professionals that actively work for infancy worldwide.

## Data availability statement

The raw data supporting the conclusions of this article will be made available by the authors, without undue reservation.

## Ethics statement

The studies involving human participants were reviewed and approved by Andalusian Biomedical Research Ethics Committee. Written informed consent to participate in this study was provided by the participants’ legal guardian/next of kin.

## Author contributions

JAI-A and RC-C: conceptualization. JAI-A, MA, and RC-C: methodology. RC-C: formal analysis, data curation, writing—original draft preparation and investigation. JAI-A and MA: resources. ICS, JAI-A, and RC-C: writing—review and editing. JAI-A: visualization and funding acquisition. ICS, JAI-A, MA, and RC-C: supervision. JAI-A, MA, and RC-C: project administration. All authors contributed to the article and approved the submitted version.

## Funding

This research was funded by the Agencia Andaluza de Cooperación Internacional para el Desarrollo, Junta de Andalucía Goverment (Spain), under the project “Estatus socioeconómico y desarrollo cognitivo en la infancia y la adolescencia: herramientas de evaluación innovadoras para poblaciones vulnerables. El caso de Guatemala,” grant number 0INN007/2017, and by Universidad Loyola Andalucía funds. Participation of ICS was also partially funded by FEDER Junta de Andalucía-Consejería de Transformación Económica, Industria, Conocimiento y Universidades/Proyecto E-SEJ-754-UGR20.

## Conflict of interest

The authors declare that the research was conducted in the absence of any commercial or financial relationships that could be construed as a potential conflict of interest.

## Publisher’s note

All claims expressed in this article are solely those of the authors and do not necessarily represent those of their affiliated organizations, or those of the publisher, the editors and the reviewers. Any product that may be evaluated in this article, or claim that may be made by its manufacturer, is not guaranteed or endorsed by the publisher.
