# Supplementary material for: ‘Mens sana in corpore Sano’: Home food consumption implications over child cognitive performance in vulnerable contexts
Source: Front Psychol. 2022 Nov 1;13:994399. doi: 10.3389/fpsyg.2022.994399 (PMC9665114; doi:10.3389/fpsyg.2022.994399)
Supplement: Supplementary file 1 [file Data_Sheet_1.docx]

Supplementary material.

Differences in cognitive performance by considering ELCSA groups in urban/rural children.

TS1. Differences between secure and severe ELCSA groups in cognitive performance.

|  | **General** | |  |  |  | **Rural** | |  |  |  | **Urban** | |  |  |  |  |
| --- | --- | --- | --- | --- | --- | --- | --- | --- | --- | --- | --- | --- | --- | --- | --- | --- |
|  | **Secure** | **Severe** |  |  |  | **Secure** | **Severe** |  |  |  | **Secure** | **Severe** |  |  |  |  |
| **Neurops. tasks** |  |  | U | *p* | g |  |  | U | *p* | g |  |  | U | *p* | g |  |
| PPVT-III | 64(91) | 38(97) | 133.5 | .196 | .52 | 68(91) | 35(97) | 74.5 | .292 | .49 | 57(46) | 54(32) | 7 | .513 | .56 |  |
| Token | 72.4(91.4) | 74.8(99) | 198 | 1.000 | .15 | 78.2(64.4) | 77.7(99) | 103 | .795 | 0 | 63.3(81.4) | 11.2(20.4) | 1 | .051 | 1.61 |  |
| Phon.Fluen./a/ | 54(54) | 36(62) | 123 | .055 | .74 | 53(51) | 40.5(42) | 81.5 | .251 | .55 | 58(52) | 16(22) | 1 | .051 | 1.99 |  |
| Sem. Fluen.ani | 54(95) | 48.5(78) | 167.5 | .439 | .3 | 54.5(82) | 53(76) | 77 | .190 | .55 | 44(91) | 55.5(33) | 8 | .641 | -.35 |  |
| SDMT | 71.3(93.6) | 47.3(90.4) | 80 | .017 | .85 | 75.3(93.6) | 47.3(86.3) | 41 | .036 | .86 | 62(90.6) | 34.2(51.9) | 4 | .231 | .93 |  |
| d2 CON | 51(94) | 41(58) | 127 | .609 | .17 | 51(86) | 41(58) | 71 | .917 | .08 | 51(62) | 51(26) | 10 | .923 | .28 |  |
| M-WCST cor. | 53(96) | 51(76) | 162 | .367 | .34 | 73(73) | 53(76) | 63.5 | .058 | .79 | 28(56) | 29(40) | 10 | .923 | .05 |  |
| M-WCST pers. | 74(92) | 48.5(81) | 112 | .027 | .7 | 78.5(67) | 48.5(81) | 37 | .002 | 1.42 | 51(76) | 35.5(65) | 11 | 1.000 | .08 |  |
| TMT-B | 70(97) | 33(89) | 85.5 | .055 | .72 | 77(75) | 47.5(89) | 42.5 | .041 | 1.12 | 11(95) | 7(12) | 5 | .533 | .57 |  |
| Stroop interf. | 38.5(96) | 45(92) | 114.5 | .847 | -.03 | 341(93) | 45(65) | 49 | .441 | -.26 | 51(84) | 32(62) | 5.5 | .436 | .43 |  |

TS2. Differences between secure and moderate ELCSA groups in cognitive performance.

|  | **General** | |  |  |  | **Rural** | |  |  |  | **Urban** | |  |  |  |
| --- | --- | --- | --- | --- | --- | --- | --- | --- | --- | --- | --- | --- | --- | --- | --- |
|  | **Secure** | **Mod.** |  |  |  | **Secure** | **Mod.** |  |  |  | **Secure** | **Mod.** |  |  |  |
| **Neurops. tasks** |  |  | U | *p* | g |  |  | U | *p* | g |  |  | U | *p* | g |
| PPVT-III | 64(91) | 63.5(100) | 584.5 | .909 | .07 | 68(91) | 63(100) | 301 | .732 | .11 | 57(46) | 69(94) | 30.5 | .479 | 0 |
| Token | 72.4(91.4) | 76.2(88) | 584 | .904 | .1 | 78.2(64.4) | 78.2(98.3) | 308 | .834 | .19 | 63.3(81.4) | 28.3(93.8) | 34 | .724 | .2 |
| Phon.Fluen./a/ | 54(54) | 50(78) | 529.5 | .556 | .06 | 53(51) | 51(75) | 294 | .784 | -.06 | 58(52) | 41(77) | 28 | .375 | .37 |
| Sem. Fluen. ani | 54(95) | 54.5(96) | 562 | .701 | -.07 | 54.5(82) | 65(96) | 293 | .621 | -.11 | 44(91) | 31(87) | 30 | .479 | .35 |
| SDMT | 71.3(93.6) | 68.4(97.7) | 460 | .485 | .19 | 75.3(93.6) | 59.4(97.7) | 204 | .197 | .37 | 62(90.6) | 81.7(75.1) | 26 | .285 | -.28 |
| d2 CON | 51(94) | 75(95) | 362.5 | .030 | -.54 | 51(86) | 69.5(95) | 183 | .054 | -.63 | 51(62) | 92(85) | 29 | .425 | -.48 |
| M-WCST cor. | 53(96) | 60(96) | 582 | .885 | -.04 | 73(73) | 66(81) | 273 | .378 | .21 | 28(56) | 31(56) | 36.5 | .860 | 0 |
| M-WCST pers. | 74(92) | 70.5(88) | 553 | .622 | 0 | 78.5(67) | 70(88) | 218 | .054 | .5 | 51(76) | 76(84) | 26 | .285 | -.44 |
| TMT-B | 70(97) | 63(93) | 400.5 | .601 | .04 | 77(75) | 65(97.7) | 207 | .221 | .37 | 11(95) | 43(67) | 16 | .622 | -.28 |
| Stroop interf. | 38.5(96) | 47(92) | 390.5 | .500 | -.17 | 341(93) | 47(88) | 220 | .466 | -.2 | 51(84) | 49(86) | 19.5 | .699 | -.16 |

TS3. Differences between secure and mild ELCSA groups in cognitive performance.

|  | **General** | |  |  |  | **Rural** | |  |  |  | **Urban** | |  |  |  |
| --- | --- | --- | --- | --- | --- | --- | --- | --- | --- | --- | --- | --- | --- | --- | --- |
|  | **Secure** | **Mild** |  |  |  | **Secure** | **Mild** |  |  |  | **Secure** | **Mild** |  |  |  |
| **Neurops. tasks** |  |  | U | *p* | g |  |  | U | *p* | g |  |  | U | *p* | g |
| PPVT-III | 64(91) | 59.5(93) | 736.5 | .273 | .27 | 68(91) | 57(93) | 285 | .180 | .37 | 57(46) | 63(87) | 98.5 | .800 | .12 |
| Token | 72.4(91.4) | 51(97.3) | 623.5 | .034 | .52 | 78.2(64.4) | 48.8(93.3) | 217 | .012 | .77 | 63.3(81.4) | 62.5(95.9) | 101.5 | .899 | .03 |
| Phon.Fluen./a/ | 54(54) | 55(76) | 815 | .698 | -.05 | 53(51) | 55(73) | 340.5 | .699 | -.13 | 58(52) | 57(76) | 98 | .800 | -.05 |
| Sem.Fluen.ani | 54(95) | 55(95) | 816.5 | .708 | .11 | 54.5(82) | 60(89) | 335 | .630 | .16 | 44(91) | 37(95) | 102 | .933 | 0 |
| SDMT | 71.3(93.6) | 54(93.2) | 527 | .017 | .57 | 75.3(93.6) | 58(93.2) | 218 | .023 | .65 | 62(90.6) | 48.2(84.1) | 85 | .420 | .39 |
| d2 CON | 51(94) | 55.5(99) | 822.5 | .930 | 0 | 51(86) | 46(85) | 305.5 | .467 | .23 | 51(62) | 69(86) | 85 | .250 | -.39 |
| M-WCST cor. | 53(96) | 53(96) | 755 | .427 | .21 | 73(73) | 66(89) | 308 | .437 | .21 | 28(56) | 17(96) | 85 | .553 | .12 |
| M-WCST pers. | 74(92) | 56(93) | 600.5 | .027 | .4 | 78.5(67) | 62.5(91) | 179.5 | .002 | .79 | 51(76) | 41(93) | 85 | .933 | -.05 |
| TMT-B | 70(97) | 70(94) | 662 | .957 | .06 | 77(75) | 81(94) | 301.5 | .953 | .15 | 11(95) | 43(89) | 85 | .344 | -.34 |
| Stroop interf. | 38.5(96) | 34(95) | 659 | .742 | -.09 | 341(93) | 30(94) | 313.5 | .977 | -.03 | 51(84) | 54(92) | 85 | .598 | -.12 |

TS4. Comparison of cognitive performance by participant ethnicity (indigenous K’iche’ vs. non-indigenous).

|  |  | **Indigenous (*n*=89)** | **Non-indigenous (*n*=61)** |  | |
| --- | --- | --- | --- | --- | --- |
| **Measures (PC)** | **n** | ***Median (SD)*** | ***Median (SD)*** | ***U*** | ***p*** |
|  |  |  |  |  |  |
| PPVT-III | 88/61 | 58.5 (28.7) | 58.7 (27.0) | 2672 | .963 |
| Token Test | 89/61 | 61.8 (30.5) | 51.1 (28.6) | 2078 | .015 |
| Phon. Fluency /a/ | 88/61 | 52.1 (17.6) | 51.1 (20.3) | 2614.5 | .788 |
| Sem. Fluency animals | 89/61 | 55.2 (26.7) | 44.2 (29.3) | 2108 | .020 |
| SDMT | 82/61 | 60.9 (27.5) | 51.8 (26.6) | 2008 | .044 |
| d2 CON | 82/61 | 54.0 (26.8) | 59.1 (27.4) | 2219.5 | .250 |
| M-WCST cor. | 88/61 | 60.7 (22.8) | 35.7 (27.4) | 1297.5 | <.001 |
| M-WCST pers. | 88/61 | 60.6 (24.9) | 47.2 (35.1) | 2178.5 | .051 |
| TMT-B | 77/49 | 64.4 (26.9) | 40.7 (32.4) | 1113 | <.001 |
| Stroop interference | 75/46 | 46.1 (29.4) | 44.7 (30.8) | 1663 | .741 |

*Note*. PC: Percentile scores (controlled for age, sex, and mean level of parental education); Phon. Fluency /a/: Phonological Fluency, sound /a/; Sem. Fluency ani.: Semantic Fluency, animals; M-WCST cor.: correct responses; M-WCST pers.: perseverative errors.
